# 609. Development of an Aspergillus Antigen Detection EIA

**DOI:** 10.1093/ofid/ofad500.675

**Published:** 2023-11-27

**Authors:** Nicole Bridges, Christopher Dailey, Andrew Hanzlicek, Joseph Wheat

**Affiliations:** MiraVista Diagnostics, Indianapolis, Indiana; MiraVista Diagnostics, Indianapolis, Indiana; MiraVista Diagnostics, Indianapolis, Indiana; MiraVista Diagnostics, Indianapolis, Indiana

## Abstract

**Background:**

Aspergillosis is a mycosis caused by *Aspergillus spp*. Invasive infections are often life threatening in individuals with weakened immune systems. Detection of *Aspergillus* galactomannan by enzyme immunoassay (EIA) is the method used most to aid in diagnose aspergillosis. Here, we present the evaluation of a new antigen detection EIA, the MiraVista® *Aspergillus* antigen detection EIA (MVD EIA) and compare it with the Platelia® *Aspergillus* EIA.

**Methods:**

Retrospective and prospective studies were conducted to evaluate the MVD EIA assay. The retrospective study consisted of bronchoalveolar lavage fluid (BALf) (n=44) from patients diagnosed with aspergillosis in accordance with EROTC guidelines. Controls(n=168) consisted of antigen negative specimens. A prospective study was conducted analyzing the agreement between the Platelia EIA and the MVD EIA. BALf and serum samples were evaluated and a receiver operator curve using Youden’s index was used to determine optimal diagnostic cutoff.

**Results:**

The retrospective study using the MVD EIA in BALf demonstrated a sensitivity of 91% in and a specificity of 88%. The prospective study demonstrated a positive agreement between the MVD EIA and the commercial Platelia EIA of 90% and 96% for BAL and serum, respectively. The negative percent agreement was 97% and 96% respectively.

**Conclusion:**

The retrospective study demonstrated that the MVD EIA is sensitive and specific when used with BALf. The prospective study demonstrated excellent agreement between the MVD EIA and the commercially available Platelia EIA. The use of this assay has the potential to aid in the diagnosis of aspergillosis.

**Disclosures:**

**Nicole Bridges, MA**, MiraVista Diagnostics: Employee **Christopher Dailey, PhD**, MiraVista Diagnostics: Employer **Andrew Hanzlicek, DVM**, MiraVista Diagnostics: employee **Joseph Wheat, MD**, MiraVista Diagnostics: Ownership Interest|MiraVista Diagnostics: Ownership Interest

